# A Transcriptional Enhancer from the Coding Region of *ADAMTS5*

**DOI:** 10.1371/journal.pone.0002184

**Published:** 2008-05-14

**Authors:** Kristen K. B. Barthel, Xuedong Liu

**Affiliations:** Department of Chemistry and Biochemistry, University of Colorado at Boulder, Boulder, Colorado, United States of America; University of Arkansas, United States of America

## Abstract

**Background:**

The revelation that the human genome encodes only ∼25,000 genes and thus cannot account for phenotypic complexity has been one of the biggest surprises in the post-genomic era. However, accumulating evidence suggests that transcriptional regulation may be in large part responsible for this observed mammalian complexity. Consequently, there has been a strong drive to locate *cis*-regulatory regions in mammalian genomes in order to understand the unifying principles governing these regions, including their genomic distribution. Although a number of systematic approaches have been developed, these all discount coding sequence.

**Methodology/Principal Findings:**

Using the computational tool PRI (Pattern-defined Regulatory Islands), which does not mask coding sequence, we identified a regulatory region associated with the gene *ADAMTS5* that encompasses the entirety of the essential coding exon 2. We demonstrate through a combination of chromatin immunoprecipitation and reporter gene studies that this region can not only bind the myogenic transcription factors MYOD and myogenin and the E-protein HEB but can also function as a very strong myogenic transcriptional enhancer.

**Conclusions/Significance:**

Thus, we report the identification and detailed characterization of an exonic enhancer. Ultimately, this leads to the interesting question of why evolution would be so parsimonious in the functional assignment of sequence.

## Introduction

Since the discovery of the SV40 enhancer, the first enhancer shown to regulate transcription of a mammalian gene [1], enhancers have been defined as DNA sequence regions that can up-regulate transcription independent of position and orientation with respect to the gene. They have been found virtually everywhere in the genome: upstream of genes, in 5′-UTRs, within introns, in 3′-UTRs, and downstream of genes [2]. They can operate from within hundreds of base pairs of the transcription start site or up to 1 Mb away [3], [4]. They can even direct transcription interchromosomally [5], [6]. There are also several reports of finding them within noncoding exons [7], [8]. However, there are very few examples of them being found within coding exons.

This does not mean that *cis*-regulatory regions cannot reside within coding exons. With rapidly expanding access to genome sequences and the development of comparative genomics tools that can locate conserved regions across genomes, researchers are increasingly relying on these methods to find regulatory regions. Unfortunately, many predictive approaches, although frequently adept at locating *cis*-regulatory regions, intentionally mask coding sequence [9]. This is probably due in large part to the higher level of conservation that would be expected in these regions, which could pose a false positive problem for identifying cis-regulatory elements using phylogenetic footprinting tools that simply look for large regions of sequence conservation.

We recently reported the development of a pattern-matching-based approach that searches for clusters of seven or more transcription factor binding sites that are absolutely conserved in order and in spacing across human, mouse, and rat genomes (http://barcode.colorado.edu/pri/) [10]. We refer to these regions as Pattern-defined Regulatory Islands (PRIs). In developing the PRI algorithm, a conscious decision was taken to not mask these sequences. Because PRI relies solely on the conservation of order and spacing of transcription factor binding sites and is insensitive to the identity of intervening bases, it is not as encumbered by the risks of highly conserved codons. Inspection of the PRI database revealed that 3.2% of the islands overlap to some degree with coding sequence. Curious about the relevance of these regions, we proceeded to test whether any of them could direct transcription in mouse C2C12 cells, a myoblast cell line in which skeletal myogenesis can be recapitulated *in vitro*. In particular, we focused on an island (termed PRI 1) associated with the gene *ADAMTS5*, which is strongly up-regulated during myogenesis. Not only does this island contain binding sites for myogenic transcription factors, it also completely encompasses an essential coding exon. We reveal in the following study that the exonic PRI 1 of *ADAMTS5* is a very potent transcriptional enhancer that depends upon the integrity of binding sites that overlap with codons.

## Results

### *ADAMTS5* Harbors a PRI that Encompasses a Coding Exon

**Figure 1:**
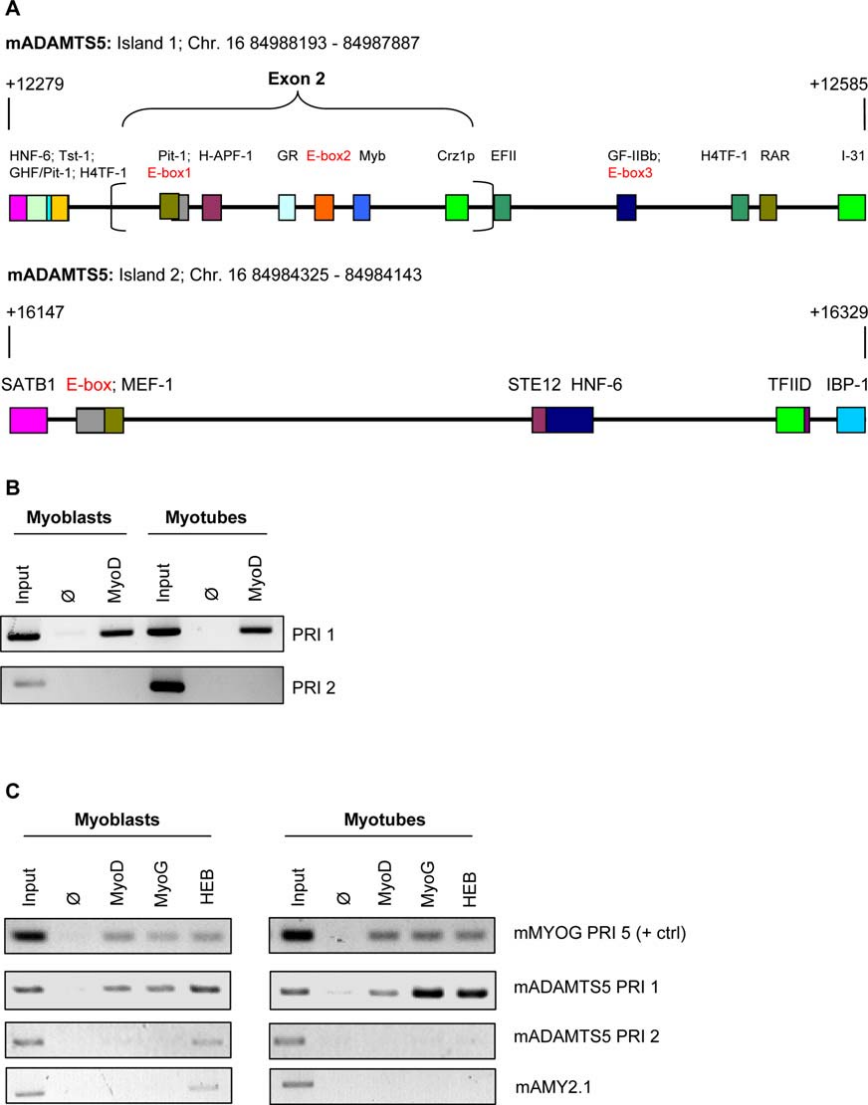
*ADAMTS5* PRI 1 Is Bound by Myogenic Transcription Factors in C2C12 Myoblasts and Myotubes. (A) Schematics of PRI 1 and PRI 2 of *ADAMTS5*. Colored boxes represent transcription factor binding sites and are labeled. E-boxes, used to find the PRIs, are labeled in red. The portion of PRI 1 that derives from exon 2 is indicated. Numbering is with respect to the start codon. (B) Chromatin immunoprecipitation using a MYOD antibody in formaldehyde-fixed, sonicated myoblast and myotube (Day 1 of differentiation) lysate indicates that PRI 1 but not PRI 2 is bound during differentiation. Input refers to 0.1% pre-immunoprecipitation input, Ø refers to no primary antibody, MYOD refers to MYOD antibody. (C) Chromatin immunoprecipitation with MYOD, MYOG, and HEB antibodies. *mMYOG* PRI 5 serves as positive control, *mADAMTS5* PRI 2 and *mAMY2.1* serve as negative controls. Input refers to 0.1% pre-immunoprecipitation input, Ø refers to no primary antibody, MYOD refers to MYOD antibody, MYOG refers to MYOG antibody, HEB refers to HEB antibody.

Annotation of the PRI database revealed that 3.2% of the assembled PRIs (22,823 out of a total 703,906 PRIs) overlap at least partially with coding sequence. In light of this figure and given our previous success with discovering that PRIs can display enhancer activity [10], we expected that it would be possible to find enhancer activity associated with exon-derived PRIs. To approach this problem, we focused on the gene *ADAMTS5*. In addition to being the aggrecanase principally responsible for the heightened degradation of the extracellular matrix molecule aggrecan seen in osteoarthritis [11], [12], its transcript is also strongly up-regulated in C2C12 cells after six days of differentiation (4.84-, 6.7-, 8.1-fold, 3 probes) [13]. We also confirmed that *ADAMTS5* is up-regulated in our C2C12 differentiation system using real-time PCR (Figure S1). We observed that two PRIs associated with this gene contain E-boxes, to which myogenic regulatory factors (MRFs) can bind, and one of these PRIs (PRI 1) encompasses the entire exon 2. Exon 2 is an essential coding exon as it contains half of the protease active site. There are seven conserved binding sites distributed throughout the exon, including two E-boxes. The other E-box-containing PRI, PRI 2, is found entirely within an intron and is approximately 3500 base pairs downstream of PRI 1. Figure 1A presents the composition of the islands in greater detail. To illustrate the broader context, the genomic locations of these PRIs mapped onto the UCSC Genome Browser (http://genome.ucsc.edu) [14], [15] are presented in Figure S2A.

It is frequently observed that there is high conservation at coding regions and lower conservation at noncoding regions (for example, see Figure S2A). Supplemental Figure 2B depicts a 10-way vertebrate Multiz alignment from Genome Browser with the PRI custom track displayed in red and the conserved binding sites overlayed on the track. In the sequence corresponding to PRI 1, not only is there high cross-species conservation associated with exon 2 (solid blue bar on the “Adamts5” track), but this conservation also extends to the flanking intronic sequence encompassed by PRI 1. Outside of the PRI region, conservation decreases noticeably. In addition, while there is strong conservation throughout the exon, there are clear dips in conservation in the intronic PRI sequence that corresponds to the spacing sequence between conserved transcription factor binding sites. This suggests that there is selective pressure for both codon preservation and binding site preservation.

**Figure 2:**
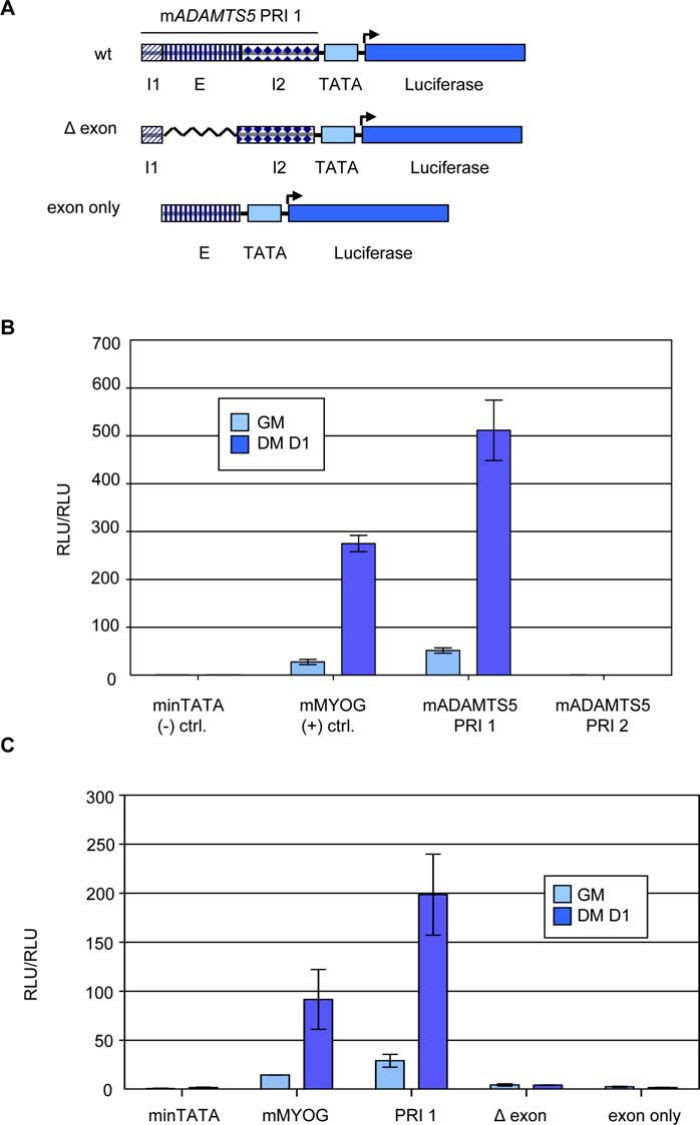
PRI 1 Drives Reporter Gene Transcription during Myogenesis and Is Dependent Upon Exon-derived Sequence. (A) Schematic of reporter gene vector construction and deletion mutants. PRIs are placed upstream of a TATA box and the firefly luciferase gene. I1 refers to sequence from Intron 1, E refers to Exon 2 sequence, I2 refers to Intron 2 sequence. (B) C2C12 cells were transfected with luciferase reporter plasmids and normalized with a renilla reporter plasmid driven by the thymidine kinase promoter. Cells were harvested as growing myoblasts (GM) and as differentiating myotubes one day after serum withdrawal (DM D1). minTATA is the background vector with a TATA box and no upstream cloned regions. Each bar represents the average of three independent experiments and error bars denote +/− SD. (C) Luciferase assay performed as in B.

### Exon-Derived PRI 1 Is Bound by Myogenic Transcription Factors during Differentiation

As an initial assessment of whether the exonic PRI 1 of *ADAMTS5* could also function as a myogenic transcriptional enhancer, we performed chromatin immunoprecipitation assays with an antibody against MYOD. Intriguingly, MYOD did bind in the region of PRI 1; in fact, the PCR signal was nearly as strong as the 0.1% input signal (Figure 1B). By contrast, the signal for MYOD binding in the vicinity of PRI 2 was virtually undetectable. We followed this result up with a more comprehensive survey of MRF and E-protein binding in C2C12 myoblasts and myotubes. E-proteins are a ubiquitous class of proteins that, amongst various other transcriptional roles, heterodimerize with MRFs to promote E-box binding and synergistic transcriptional activation [16]. HEB is the main E-protein expressed in and responsible for regulating differentiation of C2C12 cells [17], thus we selected an antibody that recognizes both α- and β-isoforms for immunoprecipitation. We also chose an antibody against myogenin to probe whether there are differences in MRF binding across this timecourse. Figure 1C depicts the results of PCR of the positive control region from *MYOG*, *ADAMTS5* PRI 1, *ADAMTS5* PRI 2, and a promoter region of amylase, AMY2-1, which has been used previously as a negative control region for MRF and HEB binding [17]. It is apparent here that while MYOD binding is still evident at PRI 1 in myoblasts and myotubes, myogenin and HEB binding dramatically increase in myotubes after one day of serum deprivation. By contrast, neither of the MRFs nor HEB binds PRI 2 at either timepoint. The observations that HEB binds PRI 1 in addition to the MRFs MYOD and myogenin and that myogenin binding appears to be regulated during differentiation indicates that one or more E-boxes within PRI 1 may truly be conveying myogenic transcriptional signals.

### PRI 1 Can Differentially Drive Transcription during Myogenesis

Although it is clear that multiple myogenic transcription factors can bind to PRI 1 of *ADAMTS5* during C2C12 differentiation, a key experiment in assessing whether this region can serve as a site of transcriptional regulation is whether it can differentially regulate reporter gene transcription. To probe this possibility, we cloned the genomic regions corresponding to PRI 1 and PRI 2 into a reporter vector upstream of a minimal TATA box-containing promoter and the luciferase gene (Figure 2A, top schematic). We then transfected the resulting constructs into C2C12 cells along with pRL-TK for transfection normalization purposes and tracked reporter gene activity in growing myoblasts (GM) and differentiating myotubes after one day of serum withdrawal (DM D1). Figure 2B demonstrates that PRI 1 robustly supports luciferase gene transcription with a 9.9-fold change, essentially equivalent to that of the *MYOG* positive control region and with slightly stronger basal level activity. Conversely, PRI 2 is incapable of enhancing transcriptional output above that of the background minimal promoter construct (minTATA), which is in line with its transcription factor binding properties. This strongly indicates that PRI 1 of *ADAMTS5* is a bona fide *cis*-regulatory region.

### The Exonic Portion of PRI 1 is Necessary for Enhancing Transcription

Having shown that PRI 1 can drive reporter gene expression and given that PRI 1 is comprised of sequence derived from both an exon and introns, an obvious question asks what the dependence on these regions is in driving transcription. Towards answering this question, we made two mutants of the wild-type PRI 1 reporter construct (Figure 2A, middle and bottom schematics). In the first (termed “Δ exon”), we deleted the exonic sequence from the middle of the island, and in the second (termed “exon only”), we deleted the intronic sequence flanking the exon. In comparing the ability of these constructs to promote luciferase gene expression during C2C12 differentiation to that of the wild-type construct (Figure 2C), it is readily apparent that both can barely support transcription above the minTATA construct and that there is absolutely no induction when differentiation is triggered. It thus appears that the exonic sequence is necessary but not sufficient to enhance transcription. The PRI hypothesis predicts this outcome as both regions contain binding sites conserved in order and in spacing.

### Both Intronic and Exonic Binding Sites Contribute to Enhancer Activity

**Figure 3:**
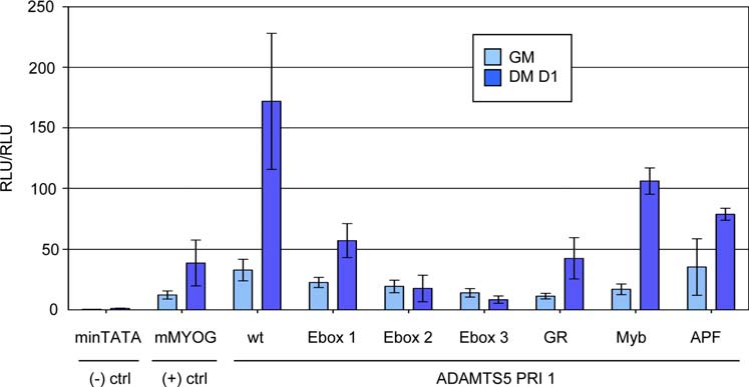
Intronic and Exonic Binding Sites Contribute to Transcriptional Output of PRI 1. (A) Luciferase results of reporter constructs of PRI 1 harboring point mutations in PRI 1 according to Table 1.

We next evaluated the contribution of individual binding sites to luciferase signal by making a number of point mutations within conserved binding sites based on already reported cases in the literature. We focused on the three E-boxes, the GR site, the Myb site, and the H-APF-1 site. The specific mutations along with the literature source for each are summarized in Table 1. We then introduced the mutant constructs into the C2C12 cells and analyzed the resultant reporter gene activity. As revealed by Figure 3, the biggest contributors to both basal level reporter gene activity and fold change during myogenesis are E-boxes 2 and 3, which are exonic and intronic respectively (numbering according to Figure 1A). E-box 1 (exonic) along with the GR site (exonic) appear to make relatively minor contributions. The effects of mutating the Myb site (exonic) and the H-APF-1 site (exonic) are rather subtle. It is worth noting that, while all three E-boxes obviously conform to the consensus site CANNTG, E-boxes 2 and 3 have the exact same sequence (CAGCTG) and contribute the most to luciferase signal while E-box 1 is different at the N positions (CATGTG). In summary, both exonic and intronic binding sites contribute to the observed PRI 1-driven reporter gene activity.

**Table 1:** Summary of binding site mutations.

Binding Site	Mutation	Reference
E-box (3)	CANNTG→CANNTa	Cheung *et al.* 2007 [10]
H-APF-1	CTGGGAA→CTGGGgA	Majello *et al.* 1990 [23]
GR	TGTTCT→TGaTCT	Dahlman-Wright *et al.* 1990 [24]
Myb	ATTGAA→ggaGAt	Nicolaides *et al.* 1991 [25]

Point mutations made for each binding studied and literature sources for these mutations.

### Assessing the Dependence on PRI Orientation

**Figure 4:**
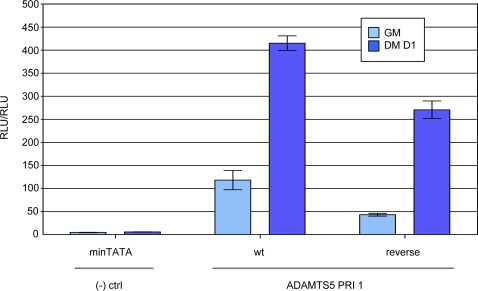
PRI Orientation Contribution to Transcriptional Output. (A) Luciferase results of reverse reporter construct of PRI 1.

The traditional definition of an enhancer is a genomic region that promotes transcription independent of position and orientation. In order to assess whether PRI 1 is independent of orientation, we reversed the orientation of PRI 1 (rPRI1) in the reporter vector. We then transiently transfected this construct along with the wild-type into the C2C12 cells and harvested Day 0 and Day 1 timepoints. In comparing to the original construct, it is apparent that, while there is an approximate 2-fold drop in signal, the fold change remains essentially the same (Figure 4). This suggests that PRI orientation may only slightly contribute to enhancer activity. It is possible that, if the PRI were moved farther away from the promoter region, orientation would be even less of a factor because there would be fewer constraints on DNA spatial arrangement such that the factors bound at the PRI could more easily be positioned with respect to the general transcription machinery.

## Discussion

Through this work, we have shown that *ADAMTS5* PRI 1 is a transcriptional enhancer that is derived in part from exonic sequence and that depends upon transcription factor binding sites, particularly E-boxes, from both the exonic and intronic portions of the island. This exon (exon 2) contains coding sequence essential to functional ADAMTS5 protein as it harbors half of the active site residues. Therefore, this is not a case of alternative start sites or noncoding exons explaining the presence of an enhancer.

How does one rationalize the dual nature of this sequence? There are a couple of potential mechanisms that could explain this co-existence. Although this island contains *ADAMTS5* exon 2, it is possible that it does not act to regulate transcription of *ADAMTS5* itself. It may instead participate in long range interactions to control another gene(s). The two flanking genes surrounding *ADAMTS5* are *ADAMTS1* and *N6amt1* (*Hemk2*). *ADAMTS1*, an aggrecanase in the same family as *ADAMTS5*, is approximately 59,000 base pairs downstream of *ADAMTS5*, but it is only up-regulated about 2-fold during myogenesis [13]. *N6amt1* (a predicted methyltransferase) is 1.5 megabases upstream of *ADAMTS5* but is expressed very poorly in C2C12 cells and changes less than 2-fold during myogenesis. While these could be targets for PRI 1-directed regulation, the possibility seems rather remote as they do not exhibit expression patterns consistent with muscle-specific genes. Still, PRI 1 may regulate some other genes more distal than these.

Alternatively, it may indeed regulate expression of *ADAMTS5*. It is worth noting that the PRI algorithm predicts another island, PRI 0, directly upstream of *ADAMTS5* (see Figure S2A for genomic position). According to the NCBI RefSeq annotation [18], PRI 0 is −79→+35 with respect to the transcription start site and −937→−823 with respect to the start codon. It harbors a conserved TATA box (−829 with respect to the start codon, +29 with respect to the RefSeq transcription start site). If PRI 1 physically interacts with PRI 0, this could provide a mechanism to explain the enhancer activity of PRI 1 in regulating transcription of *ADAMTS5* itself. Although it is counterintuitive to think that an enhancer could reside within an exon as this could present problems for the transcriptional machinery accessing this sequence, there are myriad examples of enhancers residing in introns, which would present the same problem, and yet this is obviously overcome. It is possible that a chromosomal loop forms only to enhance the initial events in transcription and that once the process has begun, the downstream sequence disengages and is free to be transcribed. Alternatively, this loop could be maintained throughout transcription but the DNA template is threaded through it as the transcript is synthesized.

One curious feature of this study is that, although there is strong differential reporter gene activity, MYOD binding does not appear to change between growing myoblasts and differentiating myotubes. The difference in activation but lack of change in MYOD ChIP could be explained by the strong recruitment of MYOG and the E-protein HEB upon the onset of differentiation. Perhaps MYOD positions the DNA and facilitates transcription by interacting with the canonical TFIID complex in myoblasts, but, having effected the necessary chromosomal arrangement, is swapped for a myogenin/HEB complex upon differentiation, which promotes even stronger transcriptional output.

Two separate E-boxes in the *ADAMTS5* exonic enhancer are required for robust transcriptional activation upon myogenic differentiation. This property is shared by the muscle-specific creatine kinase enhancer in which a pair of E-boxes has been shown to be required for cooperative binding to MYOD and subsequent transcriptional activation [19]. It has been speculated that multiple site recognition is important for decoding the concentration of MYOD and stably engaging transcription machinery [19]. Our result is consistent with this notion. Beside E-boxes, there are a number of other transcription factor binding sites in the *ADAMTS5* enhancer with invariant arrangement of spacing and orientation across human, mouse, and rat genomes. Perturbation of several binding sites can individually lead to a dramatic and nonlinear reduction in the *ADAMTS5* enhancer function (Figure 3). The requirement for the precise organization of this *cis*-regulatory element to regulate myogenic transcription suggests that this exon-encoded sequence element is a *bona fide* transcriptional enhancer.

Apart from the novelty associated with finding an exonic enhancer, the transcriptional regulation of *ADAMTS5* is an interesting topic in itself. High expression of this gene is strongly associated with osteoarthritis and mice that are homozygous for a catalytically inactive ADAMTS5 are protected against osteoarthritis when challenged with physical or chemical joint injury [11], [12]. The discovery of the role of ADAMTS5 in osteoarthritis was a major step forward in understanding the pathology of this disease and presented an obvious target for drug development. Understanding how *ADAMTS5* expression is regulated and identifying a region suitable for screening libraries of compounds for a small molecule that can inhibit transcription of *ADAMTS5* could prove very useful in the pursuit of osteoarthritis therapies. This work may provide such a region in the form of PRI 1.

The exon-derived enhancer PRI 1 is not an isolated phenomenon. Enhancer activity has been reported to be associated with exon 6 of keratin 18 [20], although the key AP-1 binding site is not evolutionarily conserved as the same region in the rat genome does not contain this site and it only leads to a three- to four-fold increase in reporter gene activity above the basal promoter. In contrast, PRI 1 elicits a 100-fold increase. We also considered another PRI that overlaps with coding sequence of the gene *IGF2*, which is up-regulated about 7-fold upon myogenic differentiation (illustrated schematically in Supplemental Figure S3A) [13]. MYOD can also occupy this region in myoblasts and myotubes as evidenced by ChIP assay (Supplemental Figure 3B). A luciferase reporter gene assay (Supplemental Figure 3C) indicates that this region possesses enhancer activity as it drives expression 22-fold above the minimal TATA reporter in the basal state, essentially equivalent to the positive control region from *MYOG* (22-fold above minimal TATA in the basal state).

In summary, we have demonstrated that *cis*-regulatory regions can overlap with coding sequence as showcased by PRI 1 of *ADAMTS5*. Moreover, we have convincing evidence that enhancer activity associated with this sequence depends upon both exon- and intron-derived transcription factor binding sites. It will be of great interest in the future to systematically determine how widespread exon-derived enhancers are throughout mammalian genomes.

## Materials and Methods

### Real-time PCR

Total RNA was prepared from C2C12 cells using an RNeasy kit (Qiagen) according to the manufacturer's instructions. It was then reverse transcribed with Transcriptor First Strand cDNA Synthesis Kit using Anchored oligo-(dT)_18_ (Roche). The real-time PCR experiment was performed using LightCycler FastStart DNA Master(PLUS) SYBR Green I following the manufacturer's instructions on a Light Cycler 2.0 (Roche). Expressions were normalized to the control gene *EPB7.2*, a gene whose expression does not significantly change during myogenesis and one we used successfully in the past [10]. The primer pair used for real-time PCR analysis of *ADAMTS5* was the same sequence reported in [12]. The primer pair used for *TNNI2* was from the Roche Universal Probe Library: 5′-AGGTGAAGGTGCAGAAGAGC; 5′-TTGCCCCTCAGGTCAAATAG.

### Plasmids and Mutagenesis

We cloned PRIs into the KpnI-PstI site of p3APP-lux in the same manner described in [21]. Point mutations and deletions were generated with the Quikchange II kit (Stratagene) per the manufacturer's instructions.

### Cell Lines and Reporter Gene Assays

C2C12 mouse myoblasts were a kind gift from Dr. Leslie Leinwand and were cultured and transfected as detailed in [10]. Luciferase assays were normalized to pRL-TK. Reporter gene assays were performed using the Dual Luciferase kit (Promega) according to the manufacturer's instructions and were read using a Dynex luminometer.

### Chromatin Immunoprecipitation

Chromatin immunoprecipitation assays were performed as described in [10]. The primers used for this analysis were designed using Vector NTI Advance 10 (Invitrogen). Primer sequences were as follows:

m*ADAMTS5* PRI 1:
5′-GTTGGGACCATATGTTCTCC
5′-CATCTGAACCAGAAGAAATCTG
m*ADAMTS5* PRI 2:
5′-GGTTGGTACCAATAATAAATATTTCACCTG
5′-AAAACTGCAGTCACTTAGCACATTTTATAA
m*AMY* 2.1 promoter (as described in [22]):
5′-TCAGTTGTAATTCTCCTTGTACGG
5′-CCTCCCATCTGAAGTATGTGGGTC
m*MYOG*
5′-TACAGGCCTTGCTCAGCTC
5′-TGTGGGAGTTGCATTCACTG

## Supporting Information

Figure S1 *ADAMTS5* Is Induced during C2C12 Differentiation. Real-time PCR analysis of mouse *ADAMTS5* and mouse *TNNI2* (positive control). Fold change in transcript level of the indicated genes relative to the control gene *EPB7.2* was determined by real-time PCR. D0, D1, and D4 refer to days of C2C12 differentiation.
(0.10 MB TIF)

Figure S2 *ADAMTS5* Harbors a PRI Encompassing Coding Exon 2. (A) A Genome Browser (http://genome.ucsc.edu) view of the mouse *ADAMTS5* gene with PRI and track displayed above. PRI predicts a regulatory region that spans Exon 2. (B) Phylogenetic conservation of PRI 1 and surrounding genomic sequence. 10-way vertebrate Multiz alignment from Genome Browser with conserved binding sites overlayed on the red PRI track.
(9.02 MB TIF)

Figure S3 A PRI Associated with *IGF2* Is a Transcriptional Enhancer. (A) Schematic of conserved binding sites in PRI 1. (B) ChIP assay reveals MYOD binding in myoblasts (D0) and myotubes (D1 after serum withdrawal). (C) Luciferase assay reveals *IGF2* PRI 1 enhances basal level transcriptional activity in C2C12 cells.
(0.71 MB TIF)
