# Supplementary figures and images for: A Transcriptional Enhancer from the Coding Region of ADAMTS5
Source: PLoS One. 2008 May 14;3(5):e2184. doi: 10.1371/journal.pone.0002184 (PMC2364661; doi:10.1371/journal.pone.0002184)

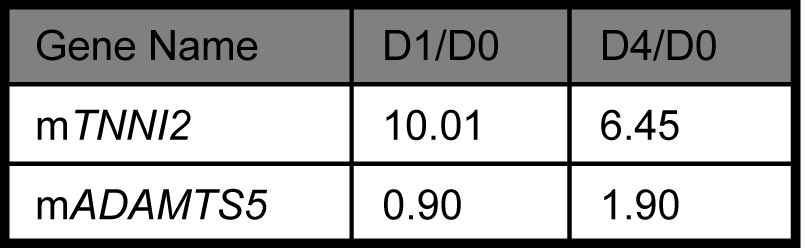

Supplement: Figure S1 — ADAMTS5 Is Induced during C2C12 Differentiation. Real-time PCR analysis of mouse ADAMTS5 and mouse TNNI2 (positive control). Fold change in transcript level of the indicated genes relative to the control gene EPB7.2 was determined by real-time PCR. D0, D1, and D4 refer to days of C2C12 differentiation. (0.10 MB TIF) [file pone.0002184.s001.tif]

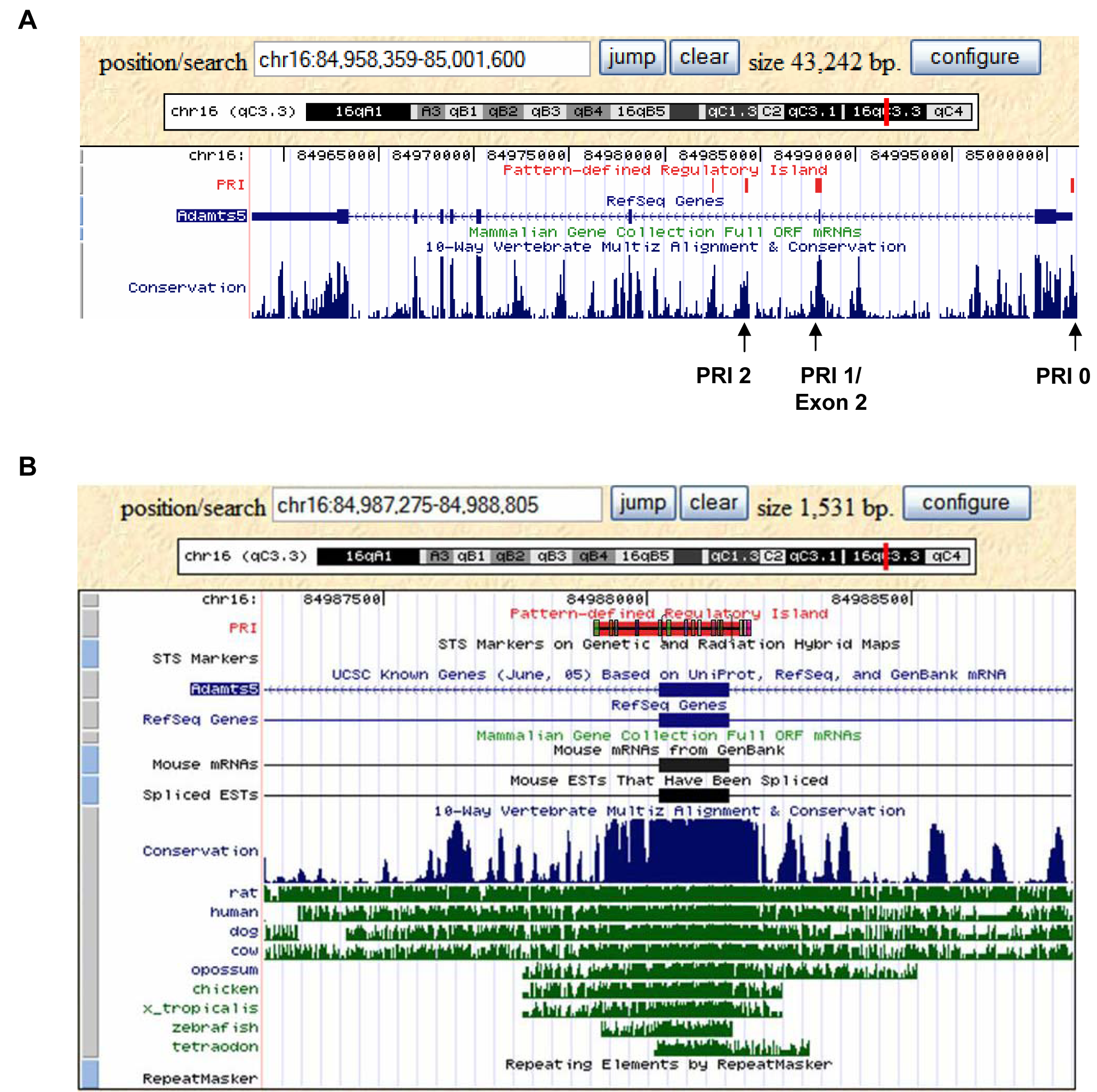

Supplement: Figure S2 — ADAMTS5 Harbors a PRI Encompassing Coding Exon 2. (A) A Genome Browser (http://genome.ucsc.edu) view of the mouse ADAMTS5 gene with PRI and track displayed above. PRI predicts a regulatory region that spans Exon 2. (B) Phylogenetic conservation of PRI 1 and surrounding genomic sequence. 10-way vertebrate Multiz alignment from Genome Browser with conserved binding sites overlayed on the red PRI track. (9.02 MB TIF) [file pone.0002184.s002.tif]

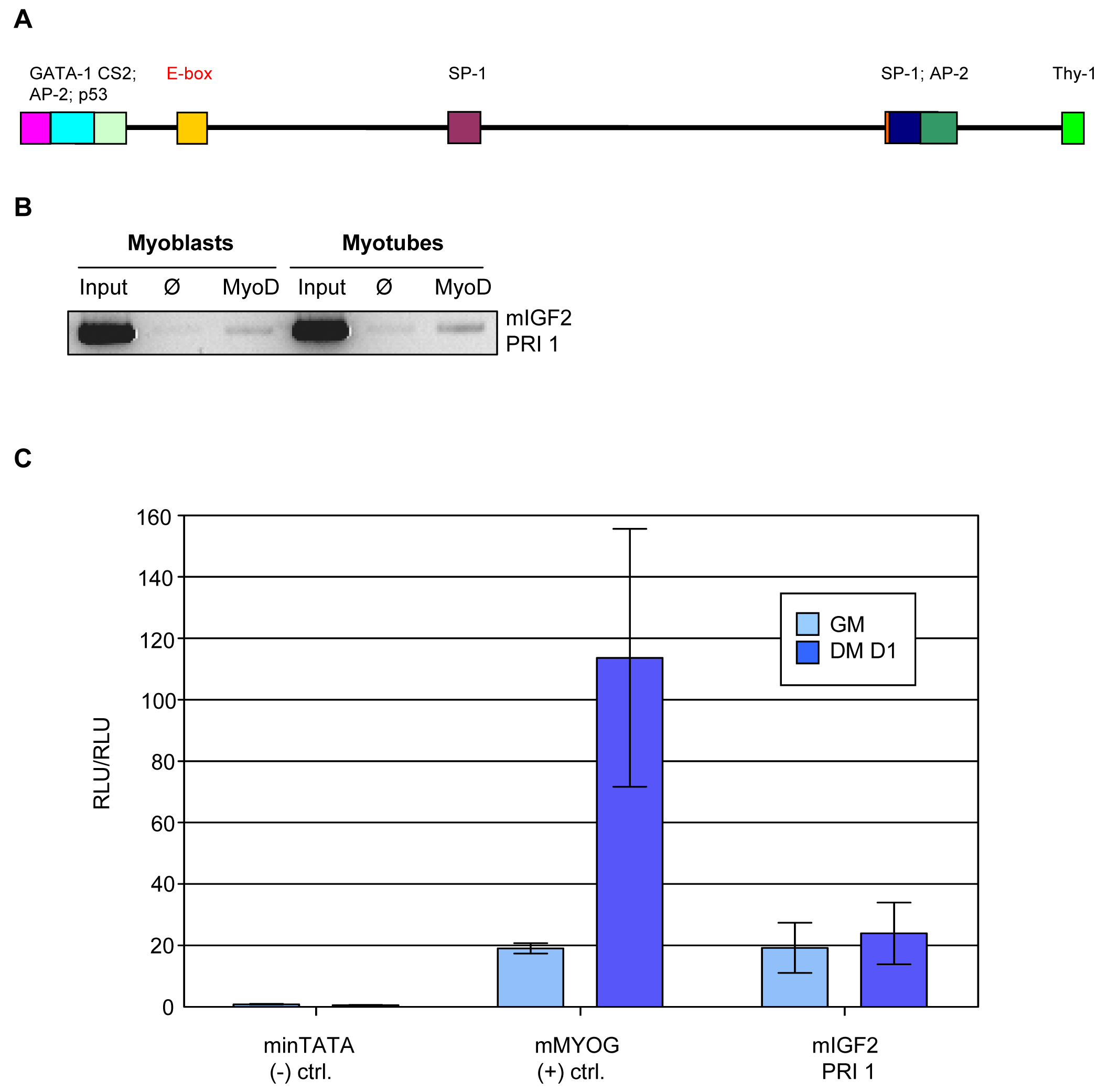

Supplement: Figure S3 — A PRI Associated with IGF2 Is a Transcriptional Enhancer. (A) Schematic of conserved binding sites in PRI 1. (B) ChIP assay reveals MYOD binding in myoblasts (D0) and myotubes (D1 after serum withdrawal). (C) Luciferase assay reveals IGF2 PRI 1 enhances basal level transcriptional activity in C2C12 cells. (0.71 MB TIF) [file pone.0002184.s003.tif]
